# Triangulation of Questionnaires, Qualitative Data and Natural Language Processing: A Differential Approach to Religious Bahá’í Fasting in Germany

**DOI:** 10.1007/s10943-023-01929-x

**Published:** 2023-10-25

**Authors:** Nico Steckhan, Raphaela Ring, Florian Borchert, Daniela A. Koppold

**Affiliations:** 1 Digital Health, Hasso Plattner Institute, University of Potsdam, 14482 Potsdam, Germany; 2 Institute of Social Medicine, Epidemiology and Health Economics, Charité-Universitätsmedizin Berlin, Corporate Member of Freie Universität Berlin, Humboldt-Universität zu Berlin, and Berlin Institute of Health, 10117 Berlin, Germany; 3 Department of Internal and Complementary Medicine, Immanuel Hospital Berlin, Berlin, 14109 Germany

**Keywords:** Religious fasting, Mindfulness, Mixed methods, Triangulation, Intermittent fasting

## Abstract

**Abstract:**

Approaches to integrating mixed methods into medical research are gaining popularity. To get a holistic understanding of the effects of behavioural interventions, we investigated religious fasting using a triangulation of quantitative, qualitative, and natural language analysis. We analysed an observational study of Bahá'í fasting in Germany using a between-method triangulation that is based on links between qualitative and quantitative analyses. Individual interviews show an increase in the mindfulness and well-being categories. Sentiment scores, extracted from the interviews through natural language processing, positively correlate with questionnaire outcomes on quality of life (WHO-5: Spearman correlation *r* = 0.486, *p* = 0.048). Five questionnaires contribute to the first principal component capturing the spectrum of mood states (50.1% explained variance). Integrating the findings of the between-method triangulation enabled us to converge on the underlying effects of this kind of intermittent fasting.

**Trial registration:**

NCT03443739

## Introduction

### Mixed Methods Including Natural Language Processing

Approaches combining qualitative and quantitative methods are increasingly being used to improve the holistic analysis of various studies, including clinical trials (Timans et al., 2019). A growth in the use of mixed methods designs has shown the value of qualitative research in medical contexts (Johnson, 2019). New methodological innovations, like natural language processing (NLP), allow important advances in the combination of research approaches in different medical domains. Natural language processing (NLP) is a methodological innovation that allows for the automated analysis of large amounts of unstructured text data, such as electronic health records, scientific publications, and social media. NLP can be used to extract valuable information from text data, including demographic information, medical conditions, symptoms, medications, and treatments. For example, NLP can be used to analyse the textual content of scientific publications, enabling researchers to identify patterns and trends in medical research. This can help researchers to identify knowledge gaps and inform the development of new research questions. NLP can be used to analyse electronic health records to identify patient populations with specific medical conditions, monitor treatment outcomes, and identify potential adverse events (Luo et al., 2017).

### Sentiment Analysis

Furthermore, sentiment analysis, which involves using natural language processing and machine learning techniques to analyse and identify the emotional tone of text, can be used to analyse patient feedback in clinical trials. This can help researchers understand how patients are responding to treatment and identify any adverse events or side effects. Also, this seems to enhance the objectivation of potentially biased qualitative analysis. This is especially helpful if mind–body interventions, mental health, or psychosomatic dimensions are considered. For tracing motivational aspects and subjective effects of lifestyle interventions, such integrative methods can also render valuable information. Therefore, the present mixed-methods approach includes interviews, questionnaires, and natural language processing (NLP) of the interview transcripts to verify findings through convergence.

### Bahá’í Fasting

A concurrent between-method triangulation, using only interview evaluation and questionnaires, has formerly been used to describe the effects of Bahá’í religious fasting (Ring et al., 2022). Followers of the Bahá’í religion follow a 19-day fast every year in March, during which, they abstain from food, fluids, and smoking during the daytime. The Bahá’í Fast (BF) can be seen as a diurnal intermittent dry fast. The Bahá’í Faith's main beliefs revolve around the concept of unity in diversity, which is reflected in the relative simplicity of rituals (Bahá’u’lláh, 2023). This is also evident in BF, where no other laws or established traditions are linked to the fasting days, allowing individuals and communities to freely decide on individual and social aspects of the fasting time. Bahá’í have a highly diverse and yet highly structured community life, with international and national institutions, as well as local agencies, facilitating communication with and between communities. The community has a global reach, with approximately 8 million followers and more than 100,000 localities in nearly every country and territory. Through this effective communication structure, as well as the fact that Bahá’í orient themselves very closely on Bahá'u'lláh's original writings, unity is highly valued in the community, and a homogeneity of practice can be assumed. Assuming that fasting is practiced uniformly within the religion, findings from Germany may be transferable to other places where BF is observed. Furthermore, the Bahá’í community holds high regard for science and scientific research based on original writings. The resulting openness to science in the Bahá’í community encourages members to participate in studies on various aspects of religious life.

In a laboratory study with a subsample of the one presented here, we were able to show that fluid balance remained stable in most individuals (Koppold-Liebscher et al., 2021), and fat metabolism was enhanced (Mähler et al., 2021). Studies have shown that religion and spirituality do influence health behaviours (Litalien et al., 2022).

Common mixed methods studies include qualitative and quantitative assessments. Integration of both quantitative and qualitative data collection and analysis techniques in a clinical context can be used to gain a more comprehensive understanding of complex health issues by combining numerical data with rich, contextual descriptions of patients' experiences, attitudes, and behaviours. An associated mixed methods study has been published by (Demmrich et al., 2021). This study investigated if religious intermittent dry fasting, in the form of Bahá'í fasting, heightens the religious experience, mindfulness, and other fasting-induced experiences.

To even further increase a comprehensive understanding of clinical phenomena, data from interviews can be input into natural language processing and quantify the sentiment while being reproducible and thus quasi-quantitative. We think that such a hybrid methodology combining the advantages of free speech with established standardized research instruments (e.g. diagnostic instruments) and computational linguistic methods like natural language processing should be developed. Therefore, this study aims to reveal the effects of an example of Bahá’í fasting using a concurrent between-method triangulation. The rationale for our proposed triangulation approach is to gain a more comprehensive and robust understanding of the effects of Bahá’í fasting on complex health issues by combining both quantitative and qualitative data collection and NLP techniques.

## Methods

### Study Design

The data originated from a longitudinal, self-controlled, observational study using a variety of laboratory (not shown here) and psychometric methods. The ethical board of Charité Universitätsmedizin Berlin approved the study protocol in January 2018 (ID: EA4/216/17) and it was registered with clinicaltrials.gov (ID: NCT03443739). Written informed consent was obtained from all participants prior to study entry and a detailed explanation of the main study design is found in our published study protocol (Koppold-Liebscher et al., 2021). The outcomes of the clinical and laboratory findings have been reported elsewhere (Koppold-Liebscher et al., 2021; Mähler et al., 2021), as have been the qualitative findings (Ring et al., 2022) and some quantitative findings (Demmrich et al., 2021).

### Qualitative Design

**Fig. 1:**
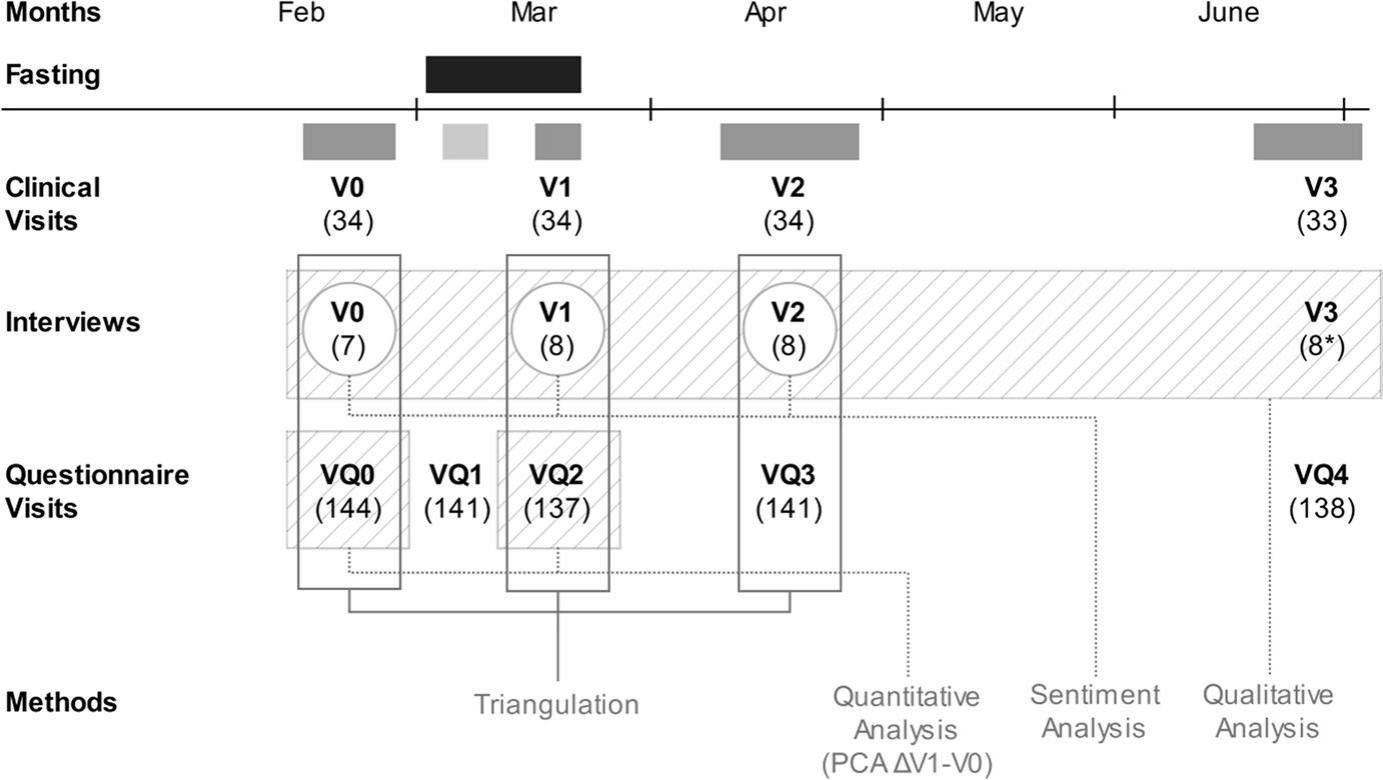
Convergent mixed methods design and alignment of qualitative and quantitative assessments. Triangulation is applied in a convergent mixed methods design, examining data from an observational trial. The connections between qualitative and quantitative analyses are based on changes from baseline principal components. Sentiment analysis is performed on the qualitative analysis data (interview transcripts), and the sentiment scores are correlated with quantitative results. V0-3: Baseline-Visit 3 (V1 (VQ2): end of the fasting period, V3 (VQ4): 3 months after baseline). Sample size in brackets. The timeline indicates the 19-day Bahá’í fasting period (1st march–20th march, in black). The grey-shaded times indicate the weeks of each visit (V0-V3). Interview transcripts of V0, V1, and V2 could be aligned with questionnaires. The PCA has been done for the delta of the baseline questionnaire (VQ0) and the immediate post-fasting questionnaire (VQ2). Here, triangulation could be applied

Semi-structured interviews and focus groups were carried out sequentially to get an unbiased picture of the effects of this type of intermittent dry fasting. At three given time points: before (*n* = 7), during (*n* = 8), and after fasting (*n* = 8). In total, 23 individual interviews were conducted. Figure 1 shows the data collection methods alongside the timeline of the interviews and sample size.

### Quantitative Design

Quantitative data collection was performed using electronic questionnaires (on virtualized machines in Charité’s demilitarized zone using Limesurvey v2). A total of 172 healthy Bahá’í volunteers were screened. Of these, 146 were considered eligible for the survey on patient-reported outcomes. The mean age was 45 ± 14 years with 35% females. Education of the subjects was mainly high with 60% graduating at a university. Because the subjects have been Bahá’í, 98.6% reported former experience with fasting. Mindfulness was measured using the Mindful Attention and Awareness Scale. Its German translation is a highly valid and reliable scale (Michalak et al., 2008). Furthermore, validated questionnaires such as the Short Depression and Happiness Scale (SDHS), Hospital Anxiety and Depression Scale (HADS), Mindful Attention and Awareness Scale (MAAS), Profile of Mood States (POMS), WHO-5 Well-being index (WHO-5), Perceived Stress Scale (PSS-10), and a self-efficacy scale (ASKU), were used.

Our data analysis methods were as follows. All questionnaires were analysed using the Wilcoxon signed-rank test, and analyses were based on an intention-to-treat dataset. Missing values were imputed using multivariate imputation by chained equations from the mice package. Post-hoc multiple comparisons were performed using Benjamini–Hochberg adjustment. We were mainly interested in short-term effects; other comparisons were published elsewhere (Ring et al., 2022). A principal component analysis of changes from baseline (V1–V0) of all questionnaires was performed. We created a principal component plot (biplot) showing all questionnaire data (ellipsoids) and the interviewed subgroup with their respective trends (arrows). All statistical analyses were done using the statistical programming language R version 3.9.

### Sentiment Analysis

We performed an automated sentiment analysis on the interview transcripts (Baseline—V2, Fig. 1). Sentiment analysis is concerned with the extraction of subjective information from text and can be applied at different levels of linguistic structures (Liu, 2012). In this research, we employed a token-level sentiment analyser on SAP HANA with access to a large lexicon of sentiment terms with known polarity. Its basic syntactic analysis detects, for example, negation (SAP, 2020). This simple approach allows the polarity of the statements to be classified on a spectrum of negative, neutral, and positive.

Based on sentiment labels automatically applied to terms in the interview transcripts, we can aggregate sentiment scores at a subject and interview level*.* For each interview transcript, the number of terms with positive and negative sentiment labels is denoted as p and n, respectively. The sentiment score *s* is given by:

$$s = \frac{p - n}{{p + n}}$$

Note that *s* ranges between − 1 and 1 and is 0 for a balanced number of positive and negative terms, meaning an overall neutral sentiment. Sentiment analysis was performed using HANA 2.0; for parsing of interview transcripts and analysing the data, we used Python 3.8, Beautiful Soup 4.8.2, and SciPy 1.7.3.

### Triangulation

Data analysis was first conducted separately, then findings were integrated from the quantitative and qualitative results, by iteratively comparing and contrasting the data (Pluye & Hong, 2014). The triangulation table assesses the convergence of the three methods by using the codes of the qualitative analysis, the PCA of questionnaire score changes from Baseline (V0-V1), and the full transcripts per subject per timepoint which were fed into the sentiment analysis (Fig. 1).

Based on these representations, cross-links between all three methods were detected. The connections between qualitative and quantitative analyses are based on changes from baseline principal components (reduced dimensions of questionnaire types). Full transcripts per subject per timepoint from qualitative analysis have been input into sentiment analysis. Sentiment scores are correlated (Spearman) with quantitative results (questionnaire scores) (Table 1).

**Table 1:** Participant demographic characteristics

	*n* = 146
	Mean (SD/%)
Age in years (mean (SD))	45.19 (13.85)
Sex = male (%)	65 (45.1)
*Education (%)*	
Still at school	0 (0.0)
Primary/secondary school graduate	4 (2.8)
Polytechnical secondary school graduate	1 (0.7)
Higher qualification secondary school graduate (Realschule)	4 (2.8)
High school graduate	34 (23.6)
Technical college or University graduate	95 (66.0)
Other	6 (4.2)
*Gross wage/year (%)*	
< 20,000 Euro	60 (41.7)
20,000–40,000 Euros	30 (20.8)
40,000–60,000 Euros	19 (13.2)
60,000–80,000 Euros	14 (9.7)
> 80,000 Euros	21 (14.6)
*Fasting experience in the past (%)*	
Yes, once	1 (0.7)
Yes, more than once	142 (98.6)
None	1 (0.7)
*Kind of fasting experienced in the past (%)*	
Prolonged therapeutic fasting	2 (1.4)
Religious fasting	138 (95.2)
Intermittent fasting	1 (0.7)
Other	2 (1.4)
Not specified	2 (1.4)
Duration of fasting experienced in the past (mean in days (SD))	18.64 (3.65)
*Frequency of fasting in the past (%)*	
Less than once a year	9 (6.3)
1–2 times per year	128 (90.1)
3–5 times per year	2 (1.4)
6–9 times per year	1 (0.7)
More than 10 times per year	2 (1.4)
*Anticipated difficulties with fasting (%)*	
Very easy	12 (8.3)
Easy	96 (66.7)
Difficult	34 (23.6)
Very difficult	2 (1.4)

## Results

### Qualitative Findings

From the interviews, we extracted three major elements that seem to play a role in Bahá’í fasting in Germany: well-being, mindfulness, and self-efficacy were the dominant topics that were represented by the selected codes. Increased introspection and reflection about their personal lifestyle triggered the behavioural changes of participants. These seem to have been further supported by the community and increased social connectedness during the fasting period. Also, the repetition of the fasting ritual seemed to increase self-efficacy. This in turn could have facilitated motivation to accomplish this fast. Prayer and meditation are important components of Bahá’í fasting and are often embedded in spiritual practices, reinforcing our assumption of increased mindfulness due to increased religiosity.

### Quantitative Findings

Results from the quantitative analysis are integrated into Table 2 showing the triangulation of all results. The positive effect on mindfulness was the most pronounced result (*p* < 0.001). Quality of life was significantly improved with fasting (WHO-5, *p* < 0.01). Stress and anxiety symptoms were also attenuated during and after fasting (PSS-10 *p* < 0.01; HADS anxiety *p* < 0.001). Measures of fatigue were decreased during the fasting period (POMS Fatigue *p* = 0.03, POMS Dejection *p* < 0.01). Non-significant effects were identified for SDHS, ASKU, POMS vigour, POMS displeasure, and HADS depression.

**Table 2:** Triangulation table showing the convergence of applied methods

Outcome	Qualitative	Quantitative		Natural language processing (Sentiment)	Convergence
	Code	Wilcoxon signed-rank test (*p*-values) V0–V1	PCA feature contribution	Spearman correlations between sentiment and questionnaire scores (rho; *p*-value)	Convergence (+) Silence (0)
Well-being	Influence well-being; Doing good to myself; to value fasting as positive; energy; lightness; Cleanse	WHO5 < **0.01** 0.59 0.15 **0.03** ** < 0.01** 0.59 ** < 0.01** ** < 0.001** 0.47	** + PC1:** WHO5 POMS Vigor SDHS **−PC1:** POMS_Fatigue POMS_Dejection POMS_Displeasure PSS-10 HADS-A HADS-D	**0.49; 0.04** 0.24, 0.35 0.08; 0.76 − 0.22, 0.38 − 0.11; 0.69 − 0.13; 0.62 − 0.42; 0.09 − 0.40; 0.11 − 0.20; 0.43	+
Mindfulness	Feeling of integration of the world; Higher awareness; focus changes; being more sensitive and empathetic; reflecting over myself and life	** < 0.001** 0.65	** + PC2:** MAAS ASKU	0.25; 0.32 0.09; 0.72	+
Discipline and freedom	Freedom; Challenges; Discipline; Development				0
Behavioural change	Assistive preparations for fasting; To create new habits; To eat mindfully				0

Bold values indicate statistical significance

*PCA* principal component analysis; + *PC1* positive effect contribution to principal component 1; − *PC1* negative effect contribution to principal component 1; − *PC2* negative effect contribution to principal component 2 (coordinate directions are inversed order)

**Fig. 2:**
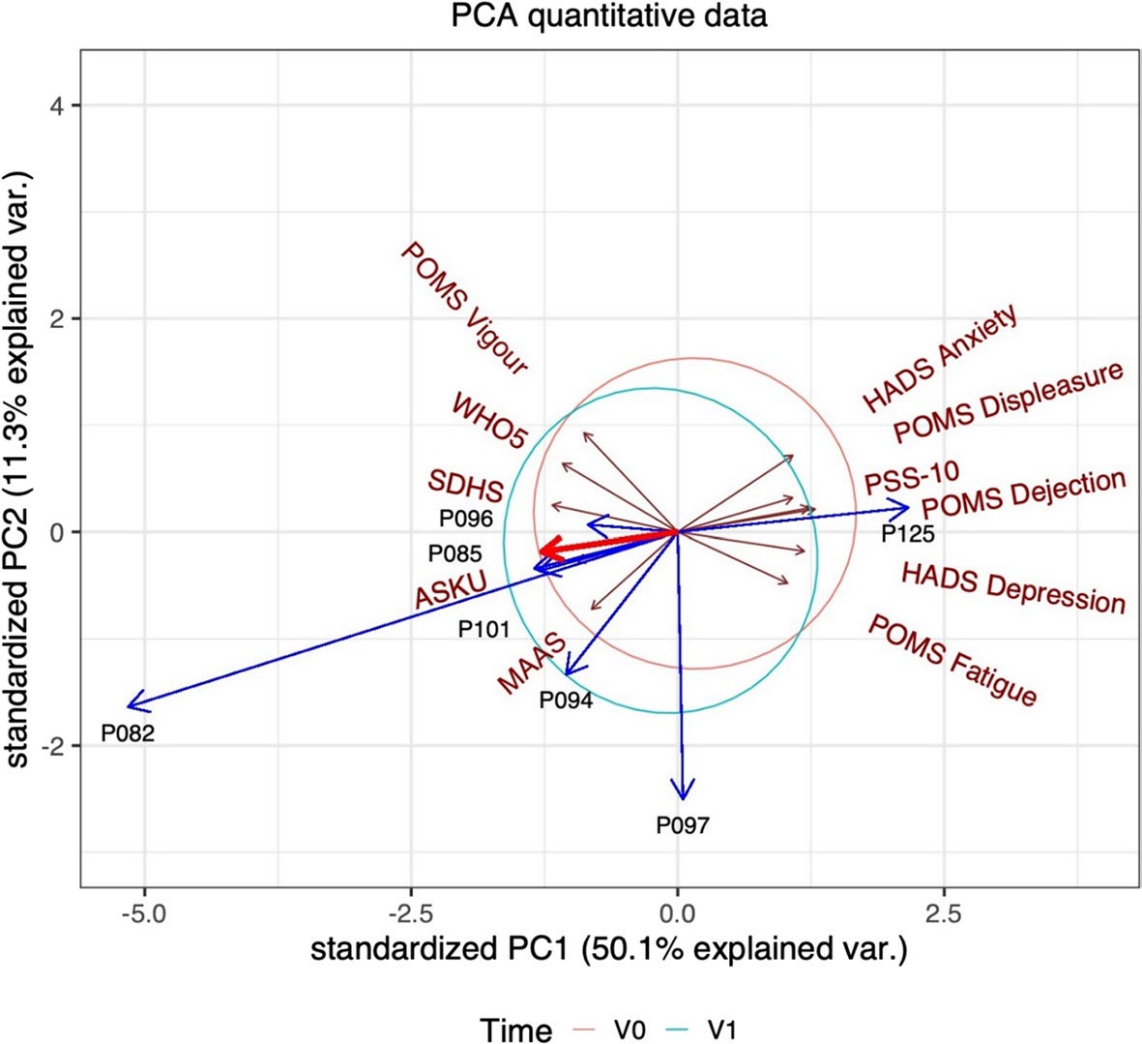
Principal component plot with changes from baseline. Explanation of Fig. 2 is as follows: Ellipsoids represent the normal 68% confidence per timepoint. Blue arrows: Changes from individual baseline of interview partners. Brown arrows: correlation between the original variable and the principal component; Red arrow: time trend; ASKU: self-efficacy scale; HADS: Hospitality Anxiety and Depression scale; MAAS: Mindful Attention and Awareness Scale; POMS: Profile of mood states; PSS-10: Cohen’s perceived stress scale; SDHS: Short Depression and Happiness Scale; WHO-5: WHO-Five Well-being index

The principal component plot (Fig. 2) shows the trend of the psychometric data from baseline (origin) to V1 (end of blue arrows). This shift reflects an increased effect on well-being and mindfulness, while features like depression, stress, and fatigue were less pronounced with fasting. The first principal component captured 50.1% explained variance. WHO-5, SDHS, ASKU, and MAAS are in one direction. In the opposite direction, POMS Fatigue, POMS Dejection and Depression, PSS-10, HADS-Depression can be seen.

The ellipsoids represent a full sample of 146 subjects. The interviewed subgroup (*N* = 7) shows the same trend between Baseline and V1 (thick red arrow). Interestingly, subject P125 seems to be situated in an opposite direction (decreased well-being) under all three methods, supporting the reliability of our approaches.

### Sentiment Analysis

We calculated sentiment scores for a total of 20 interview transcripts for 7 subjects, resulting in a total of 1670 sentiment mentions or a mean of 83.5 labeled terms per interview (SD = 31.98). The mean average sentiment score over all interviews is 0.634 (SD = 0.19), indicating an overall positive sentiment (the expected value is 0).

Sentiment scores at the individual level at a given time point are positively correlated with WHO-5 (Spearman correlation *r* = 0.486, *p* = 0.048). There are no significant correlations with other questionnaires (Table 2).

### Triangulation

By combining all three methodological investigations, we identified two common themes: well-being and mindfulness, of which the former seems to have the highest convergence between approaches. Here, positive effects and time trends were present in all respective outcomes. WHO-5, POMS vigour, SDHS positively contributed to the first subdimension (principal component 1) of the feature reduction. However, POMS fatigue, dejection, displeasure, PSS-10 and both HADS subscores, are negatively associated with PC1. Interestingly, changes in WHO-5 were positively correlated with the sentiment score. Other correlations between sentiment scores and questionnaires were not significant after adjustment. The second qualitative category of mindfulness (principal component 2) was identified, where MAAS and ASKU also contributed to this dimension. Further qualitative categories like discipline, freedom, and behavioural change could not be directly linked to the quantitative methods.

## Discussion

Based on our study, Bahá’í Fasting (BF) appears to have a positive effect on well-being and stress reduction, which may be partly attributed to the stress-reducing effects of mindfulness, anti-depressive and anxiolytic effects. Additionally, the increased religiosity and changes in health-related behaviour reported by participants during fasting may have contributed to this effect.

Well-being is a multifaceted construct that includes physical, mental, emotional, social, and spiritual aspects. Our study found that BF targets all these aspects, as participants reported feeling physically well with their altered daily habits during fasting, while mindfulness during fasting improved their mental well-being. The first principal component represents these aspects and summarizes the overall positive sentiment and health effects which could be identified with support of natural language analysis in the interview transcripts. Our results are in line with previous studies on Ramadan fasting, which were found to have a positive influence on stress, anxiety, and depression. A systematic review and meta-analysis on the effects of fasting on stress, anxiety, and depression suggest lifestyle factors such as sleep deprivation as a possible reason (Berthelot et al., 2021).

By integrating three methodological approaches, we were able to converge the categories of the qualitative analysis: well-being and mindfulness featured prominently in the questionnaires. The first two principal components could be aligned with themes from the qualitative analysis. Also, the questionnaires that contributed to the components were consistently associated with positive and negative directions. The first component reflected the spectrum of well-being-related questionnaires from stress and depression to positive mood and well-being. This indicates a reverse relationship between extreme cases that were present in our subsample and is known from other studies (Kessler et al., 2018).

Sentiment analysis of interview transcripts confirms the results from the WHO-5 questionnaire, which we expected because sentiment analysis captures positive or negative expressions. The observed increase in well-being and mindfulness is consistent with current research on the positive correlation of mindfulness, self-efficacy, and well-being (Schönfeld et al., 2016). Studies on Ramadan fasting also report associations of fasting with increased well-being (Akbari et al., 2022). Also, religiosity and spirituality influence well-being positively (Majda et al., 2022; Litalien et al., 2022). The stress-reducing effect of mindfulness due to its antidepressant and anxiety-relieving effects (Luszczynska et al., 2005) combined with a focus on religiosity and spirituality during fasting, which are resilience-promoting factors (Foureur et al., 2013), may explain our finding of increased well-being. The quantitative analysis reproduced the beneficial effects that were discussed during the interviews.

## Limitations

A limitation of the study is that qualitative and quantitative analysis were conducted in parallel; it is recommended that the qualitative assessment is conducted before quantitative methods are applied in sequential order (Pluye & Hong, 2014). However, this was not possible in our study setting; we used a concurrent between-method triangulation. Detection of sentiments based on an emotion lexicon and simple syntactic analysis is known to be error-prone, resulting in some noise in the sentiment scores. While state-of-the-art approaches for sentiment analysis are based on machine learning from large text corpora, these are not readily applicable in our setting, due to the lack of large, annotated training data for the German language and in particular for the domain of transcribed interviews.

## Future Directions

A promising direction for future research will be the deeper integration of NLP technology into qualitative data analysis, in particular, considering classification or information extraction problems beyond simple binary sentiment analysis. NLP methods can be implemented for detecting categories like behavioural change and discipline, or symptoms like stress, depression, and anxiety; this will enable a more complete triple triangulation in the future. However, these methods currently require corpora that have been annotated for these phenomena, which are still scarce for most concepts and languages other than English. Sentiment analysis can be a helpful tool in mixed methods studies because it allows researchers to quantitatively analyse the emotional tone of textual data, such as survey responses, open-ended interview questions, or social media posts (Denecke & Deng, 2015). This can provide a more nuanced understanding of the attitudes and opinions expressed by participants in the study, and complement the qualitative data collected through other methods such as interviews or focus groups. Sentiment analysis can also help researchers identify patterns and trends in the data that might be missed through manual coding or analysis. For example, sentiment analysis can be used to identify common themes or concerns across a large sample of survey responses, or to track changes in attitudes over time in longitudinal studies. Another advantage of sentiment analysis is that it can be used to analyse unstructured data, which can be difficult to quantify using traditional methods. By using sentiment analysis to assign a numerical value to the emotional tone of text, researchers can more easily compare and contrast different types of data, such as social media posts and survey responses.

Overall, sentiment analysis can be a valuable tool in mixed methods studies, allowing researchers to analyse large amounts of textual data in a systematic and quantitative way, while also providing insights into the emotional tone and attitudes expressed by study participants.

## Conclusions

The study investigated the effects of Bahá’í fasting on well-being, mindfulness, and self-efficacy among a sample of Bahá’í participants in Germany. Using a concurrent between-methods triangulation, we were able to converge categories and foster a holistic understanding of the impact of religious fasting. The ability to use natural language processing and sentiment analysis with qualitative analysis could bridge and underline an innovative quasi-quantitative multidisciplinary approach.

## Data Availability

Data described in the manuscript will be made available upon request pending application and approval.
